# Time Spent Eating, by Immigrant Status, Race/Ethnicity, and Length of Residence in the United States

**DOI:** 10.5888/pcd17.200122

**Published:** 2020-11-25

**Authors:** Jim P. Stimpson, Brent A. Langellier, Fernando A. Wilson

**Affiliations:** 1 Drexel University, Dornsife School of Public Health, Philadelphia, Pennsylvania; 2 University of Utah, Matheson Center for Health Care Studies, Salt Lake City, Utah

## Abstract

**Introduction:**

Time spent eating is associated with obesity and diet-related diseases. We examined the association between time adults spent eating, immigrant status, race/ethnicity, and race/ethnicity among adults in the United States.

**Methods:**

We used multivariate linear regression to analyze a cross-sectional, nationally representative sample of respondents aged 19 years or older (N = 192,486) from the 2016 American Time Use Survey. The outcome measures were time spent per day on primary eating and drinking and secondary eating. The predictors were immigrant status, race/ethnicity, and years spent living in the United States.

**Results:**

Multivariate adjusted minutes per day spent on primary eating and drinking were 66.4 for noncitizens, 66.5 for naturalized citizens, and 60.1 for US-born individuals. Multivariate adjusted minutes per day spent on secondary eating were 11.1 for noncitizens, 12.2 for naturalized citizens, and 12.9 for US-born individuals. Minutes per day spent on primary eating and drinking for immigrants by length of residence in the United States was 69.7 minutes for 5 years or less of residence, 67.9 minutes for 6 to 10 years of residence, 63.6 minutes for 11 to 15 years of residence, and 63.6 minutes for more than 15 years of residence. Minutes per day spent on secondary eating for immigrants by length of residence was 5.5 minutes for 5 years or less of residence, 9.7 minutes for 6 to 10 years of residence, 8.4 minutes for 11 to 15 years of residence, and 12.6 minutes for more than 15 years of residence.

**Conclusion:**

Time spent eating varied by immigrant status and length of residence in the United States.

**Summary**

**What is already known on this topic?**

Obesity risk increases with time spent in the United States, and time spent in the United States is negatively associated with diet quality and positively associated with energy intake and consumption of pre-prepared or processed foods.

**What is added by this report?**

An understudied but potentially important mechanism to explain the change in eating behaviors of immigrants and their offspring is change in time spent in meal preparation and consumption. We present data on immigrant status by nativity, citizenship, race/ethnicity, and length of residence in the United States.

**What are the implications for public health practice?**

Immigrants may be shifting time toward eating behaviors characterized by less time spent food shopping, preparing meals, and food consumption as a primary activity and more time spent eating while engaged in other activities such as watching television. Public health policy and programs should address the negative impact that acculturation may have on eating behaviors.

## Introduction

Time spent eating is associated with obesity and diet-related diseases (1,2). An emerging literature suggests several likely pathways in which time spent preparing and eating meals and snacks affects diet quality and energy intake (3–5). Meals prepared in the home from scratch tend to have fewer calories and to be generally more nutritious than processed foods and food eaten away from home (eg, restaurant meals) (6–9). The context in which meals are eaten also matters. Eating while engaging in sedentary behavior such as television viewing has been linked to higher calorie consumption and weight gain (10,11).

Collectively, data from time use diaries suggest a trend over time toward more pre-prepared or semi-prepared foods and less time eating and drinking but that the association is modified by demographic characteristics, such as race/ethnicity (3–5,12). Racial/ethnic disparities in obesity have been linked to time used in meal preparation, food consumption, and snacking during sedentary behavior (13–15). Evidence from intervention studies suggests that culturally tailored approaches are more effective at improving nutrition behaviors and improved health outcomes (13–15).

Obesity risk increases with acculturation and time spent in the United States (16). Although the dietary patterns of immigrants are nuanced and vary by subgroup, studies have generally found that acculturation and time spent in the United States are negatively associated with diet quality and positively associated with energy intake and consumption of processed foods (17–22). Much of the literature investigating the link between acculturation, diet, and obesity has focused on the roles of obesogenic environments (22). An understudied but possible mechanism to explain the change in diets of immigrants and their offspring during the acculturation process and in increasing time spent in the United States is changes in meal preparation and consumption patterns (23,24). Specifically, immigrants may shift toward patterns characterized by less time spent with meal preparation of fresh foods and faster consumption times from pre-prepared or processed foods (25–27).

Our objective was to understand the association between time spent eating, immigrant status, and race/ethnicity among adults in the United States. Time spent eating was defined by primary eating and drinking, and secondary eating. Primary eating time was defined as time spent on shopping, meal preparation, and consumption of food as the primary activity. Secondary eating was defined as time spent eating while engaged in another activity, such as a sedentary behavior like watching television. Based on the literature of obesity, food behavior, and time use, we hypothesized that acculturation of immigrants would be negatively associated with primary eating time and positively associated with secondary eating time. We also hypothesized that we would find differences in eating time by immigrant status and race/ethnicity.

## Methods

We used cross-sectional, nationally representative data from the 2016 American Time Use Survey (ATUS) provided by the University of Minnesota’s Integrated Public Use Microdata Series (28). ATUS is a time diary telephone survey of the United States that asks 1 noninstitutionalized respondent aged 15 years or older from each sampled household to provide detailed information on the time spent on various activities in a 24-hour period from 4:00 AM the day before the interview to 4:00 AM of the interview day. The ATUS sample is drawn from households that completed the Current Population Survey interview fielded by the US Census Bureau; data include time diary, demographic, labor force participation, and household information. More details about ATUS, including the user’s guide, questionnaire, and list of published research papers, can be found on the US Bureau of Labor Statistics website (28).

We used the most recent data available from the ATUS Eating and Health Module, which is collected periodically and sponsored by the Economic Research Service of the US Department of Agriculture (28). The Eating and Health Module adds supplemental questions to ATUS such as time spent eating and drinking, grocery shopping and fast food purchase, meal preparation, food sufficiency, food assistance, height, weight, general health, and exercise. Sample weights supplied by the US Census Bureau and applied to the individual ATUS and Eating and Health Module respondent data produced nationally representative estimates for an average day in 2016. After listwise deletion of 6,356 respondents with missing data, the final analytic sample size was 192,486 adults aged 19 years or older with complete information on all study variables.

The outcome measures were time spent per day in primary eating and drinking and time spent per day in secondary eating measured in minutes. Generally, primary eating and drinking time is the total amount of time during the diary day that the respondent spent in primary eating and drinking, which includes shopping, meal preparation, and meal consumption (1–5). This variable is calculated by summing the amount of time in minutes the respondent spent in eating and drinking as a primary activity. Secondary eating is eating that occurred during a primary activity and is calculated as the sum of all time in minutes during the diary day that the respondent spent in secondary eating. The specific question text for primary and secondary eating was as follows: “We’re interested in finding out more about how people fit meals and snacks into their schedules. Yesterday, you reported eating or drinking between [fill in times from respondent’s time diary]. Were there any other times you were eating yesterday — for example, while you were doing something else? About how long would you say you were eating while you were [fill in activity]?” (28) The continuous measurement for these outcomes was truncated at 121 minutes per day to normalize the distribution.

The predictor variables were immigrant status, which was derived from nativity status and citizenship status (born in the United States, naturalized citizen, noncitizen), years spent living in the United States (0–5, 6–10, 11–15, >15 years), and race/ethnicity (White non-Hispanic, Hispanic, Black non-Hispanic, Asian non-Hispanic, other race/ethnicity). Control variables were age (19–25, 26–44, 45–64, 65–85), sex (male, female), marital status (currently married, previously married, never married), presence of children younger than 18 years in the household (yes, no), immigrant status (US born, naturalized citizen, noncitizen), education (less than high school, high school diploma, some college/associate’s degree, bachelor’s degree, graduate degree), employment status (employed, not employed), family income (<$20,000; $20,000–$39,999; $40,000–$74,999; $75,000–$149,999; ≥$150,000), residence in a metropolitan area (yes, no), region of country residence (Northeast, Midwest, South, West), and perceived health (excellent/very good/good, fair/poor).

We used multivariate linear regression models because the outcome measure was measured continuously as minutes per day. We calculated the predicted average number of minutes spent per day in primary and secondary eating by immigrant status and race/ethnicity. All analyses were conducted using Stata version 16 (StataCorp LLC) and accounted for survey weights and complex survey design to produce nationally representative estimates. We set 2-sided significance at *P* < .05.

## Results

Overall, 84.5% of the sample was born in the United States, 7.5% were naturalized citizens, and 8.0% were noncitizens (Table 1). Compared with US-born respondents, there was a lower proportion of naturalized citizens aged 19–25 (5.2%) and a greater proportion of naturalized citizens aged 45–64 (43.8%). Compared with US-born respondents, there was a lower proportion of noncitizens aged 19–25 (8.8%), 45–64 (25.0%), and 65–85 (5.8%) but nearly double the proportion of adult noncitizens aged 26–44 (60.4%). We found no significant sex differences by immigrant status. 

**Table 1:** Weighted Characteristics of Adults Aged 19 Years or Older (N = 192,486), by Immigrant Status, American Time Use Survey, 2016^a^

Characteristic	US Born	Naturalized Citizen	Noncitizen
	% (95% CI)		
**Immigrant status**	84.5 (84.3–84.8)	7.5 (7.3–7.7)	8.0 (7.8–8.1)
**Age, y**			
19–25	11.7 (11.4–12.0)	5.2 (4.6–6.0)	8.8 (8.0–9.7)
26–44	31.6 (31.3–31.9)	30.8 (29.8–31.8)	60.4 (59.3–61.5)
45–64	35.2 (34.9–35.5)	43.8 (42.7–44.9)	25.0 (24.1–25.9)
65–85	21.6 (21.3–21.8)	20.2 (19.4–21.0)	5.8 (5.4–6.2)
**Sex**			
Male	43.9 (43.5–44.2)	43.9 (42.8–45.0)	43.4 (42.4–44.5)
Female	56.1 (55.8–56.5)	56.1 (55.0–57.2)	56.6 (55.5–57.6)
**Marital status**			
Never married	25.2 (24.9–25.5)	16.5 (15.6–17.4)	23.0 (22.0–24.0)
Currently married	55.9 (55.5–56.2)	71.8 (70.8–72.8)	66.8 (65.8–67.9)
Previously married	19.0 (18.7–19.2)	11.7 (11.2–12.3)	10.2 (9.7–10.8)
**Children younger than 18 years in the household**			
No	65.4 (65.1–65.7)	54.5 (53.4–55.6)	38.9 (37.9–40.0)
Yes	34.6 (34.3–34.9)	45.5 (44.4–46.6)	61.1 (60.0–62.1)
**Race/ethnicity**			
White, non-Hispanic	77.3 (77.1–77.6)	24.8 (23.9–25.8)	14.5 (13.7–15.4)
Hispanic	9.0 (8.8–9.2)	36.9 (35.9–38.0)	58.8 (57.7–59.9)
Black, non-Hispanic	10.8 (10.6–11.0)	15.9 (15.1–16.8)	7.0 (6.5–7.6)
Asian, non-Hispanic	1.1 (1.0–1.2)	21.5 (20.6–22.4)	18.6 (17.8–19.5)
Other race/ethnicity, non-Hispanic	1.7 (1.6–1.8)	0.8 (0.7–1.0)	1.0 (0.8–1.3)
**Education**			
Less than high school	5.9 (5.8–6.1)	14.9 (14.0–15.8)	31.5 (30.5–32.4)
High school diploma	28.7 (28.4–29.0)	28.2 (27.2–29.3)	23.1 (22.2–24.0)
Some college/associate’s degree	28.4 (28.1–28.7)	21.3 (20.5–22.2)	15.7 (14.8–16.6)
Bachelor’s degree	23.1 (22.8–23.4)	20.5 (19.7–21.4)	15.7 (15.0–16.5)
Graduate degree	13.9 (13.7–14.1)	15.0 (14.3–15.7)	14.0 (13.3–14.8)
**Employment status**			
Not employed	36.0 (35.6–36.3)	34.4 (33.4–35.5)	33.0 (32.0–34.0)
Employed	64.1 (63.7–64.4)	65.6 (64.5–66.6)	67.0 (66.0–68.0)
**Family income, $**			
<20,000	13.1 (12.9–13.3)	11.6 (10.9–12.3)	19.6 (18.8–20.3)
20,000–39,999	18.9 (18.6–19.1)	20.1 (19.1–21.0)	30.0 (29.1–31.0)
40,000–74,999	28.0 (27.8–28.3)	28.5 (27.6–29.5)	24.4 (23.5–25.4)
75,000–149,999	28.7 (28.4–29.0)	24.9 (23.9–25.9)	15.0 (14.2–15.8)
≥150,000	11.3 (11.1–11.5)	15.0 (14.2–15.8)	11.1 (10.2–11.9)
**Metropolitan residence**			
No	17.0 (16.8–17.2)	4.9 (4.3–5.5)	5.6 (5.2–6.0)
Yes	83.0 (82.8–83.2)	95.2 (94.5–95.7)	94.4 (94.0–94.8)
**Region of country**			
Northeast	16.6 (16.4–16.9)	25.3 (24.3–26.3)	17.6 (16.8–18.4)
Midwest	25.6 (25.3–25.9)	14.5 (13.7–15.3)	12.6 (11.9–13.3)
South	37.2 (36.8–37.5)	30.7 (29.7–31.8)	42.6 (41.5–43.7)
West	20.7 (20.4–20.9)	29.5 (28.5–30.5)	27.2 (26.3–28.1)
**Perceived health**			
Excellent/very good/good	85.0 (84.8–85.3)	84.1 (83.3–84.9)	83.9 (83.1–84.6)
Fair/poor	15.0 (14.8–15.2)	15.9 (15.1–16.7)	16.1 (15.4–16.9)
**Mean min/d spent in primary eating and drinking**	60.2 (60.0–60.4)	67.3 (66.4–68.1)	64.5 (63.8–65.2)
**Mean min/d spent in secondary eating**	13.0 (12.8–13.2)	11.5 (11.1–12.0)	10.2 (9.7–10.7)

a Primary eating and drinking consists of the total amount of time during the diary day that the respondent spent in eating and drinking as a primary activity. Secondary eating is the sum of all time during the diary day that the respondent spent eating during a primary activity.

We found differences in the proportion of racial/ethnic identification by immigrant status. Among US-born respondents, 77.3% reported their race/ethnicity as White, non-Hispanic; 9.0% reported Hispanic ethnicity; 10.8% reported their race/ethnicity as Black, non-Hispanic; 1.1% reported their race/ethnicity as Asian, non-Hispanic; and 1.7% reported their race/ethnicity as other non-Hispanic race/ethnicity. Among naturalized citizens, 24.8% reported their race/ethnicity as White, non-Hispanic; 36.9% reported Hispanic ethnicity; 15.9% reported their race/ethnicity as Black, non-Hispanic; 21.5% reported their race/ethnicity as Asian, non-Hispanic; and 0.8% reported their race/ethnicity as other non-Hispanic race/ethnicity. Among noncitizens, 14.5% reported their race/ethnicity as White, non-Hispanic; 58.8% reported Hispanic ethnicity; 7.0% reported their race/ethnicity as Black, non-Hispanic; 18.6% reported their race/ethnicity as Asian, non-Hispanic; and 1.0% reported their race/ethnicity as other non-Hispanic race/ethnicity. The average time spent in a day in 2016 on primary eating and drinking was 60.2 minutes for US-born respondents, 67.3 minutes for naturalized citizens, and 64.5 minutes for noncitizens. (The detailed, unadjusted relationship between the outcome measures and immigrant status and race/ethnicity are available on request from the corresponding author.)

Results of multivariate linear regression for primary eating and drinking and secondary eating time per day indicated that noncitizens spent, on average, 66.4 minutes (95% CI, 65.6–67.2) compared with 66.5 minutes (95% CI, 65.5–67.4) for naturalized citizens and 60.1 minutes (95% CI, 59.9–60.3) for US-born respondents (Table 2). We found a significant difference in primary eating and drinking time between noncitizens and US-born respondents and between naturalized citizens and US-born respondents. However, we did not find a significant difference between noncitizens and naturalized citizens in primary eating and drinking time. (Full linear regression results for all variables included in the model are available on request from the corresponding author.)

**Table 2:** Adjusted Mean Number of Minutes Per Day Spent in Primary Eating and Drinking and Secondary Eating Activities Among Respondents Aged 19 or Older (N = 192,486), by Immigrant Status and Race/Ethnicity American Time Use Survey, 2016^a^

Characteristic	Primary Eating and Drinking	Secondary Eating
	Mean (95% CI)	
US born	60.1 (59.9–60.3)	12.9 (12.7–13.0)
Naturalized citizen	66.5 (65.5–67.4)	12.2 (11.6–12.8)
Noncitizen	66.4 (65.6–67.2)	11.1 (10.4–11.8)
US born, White non-Hispanic	61.8 (61.5–62.1)	12.9 (12.7–13.1)
US born, Hispanic	59.0 (58.4–59.7)	12.7 (12.2–13.3)
US born, Black non-Hispanic	48.7 (48.1–49.3)	13.0 (12.6–13.5)
US born, Asian non-Hispanic	69.6 (68.4–70.7)	11.6 (10.7–12.5)
US born, other non-Hispanic race/ethnicity	51.0 (49.6–52.4)	13.9 (12.8–15.0)
Naturalized citizen, White non-Hispanic	68.2 (67.2–69.2)	12.2 (11.5–12.9)
Naturalized citizen, Hispanic	65.4 (64.5–66.3)	12.0 (11.4–12.6)
Naturalized citizen, Black non-Hispanic	55.1 (53.9–56.2)	12.3 (11.7–13.0)
Naturalized citizen, Asian non-Hispanic	76.0 (74.8–77.1)	10.9 (10.1–11.7)
Naturalized citizen, other non-Hispanic race/ethnicity	57.4 (55.7–59.1)	13.2 (11.9–14.5)
Noncitizen, White non-Hispanic	68.1 (67.2–69.0)	11.2 (10.4–11.9)
Noncitizen, Hispanic	65.4 (64.5–66.2)	11.0 (10.4–11.5)
Noncitizen, Black non-Hispanic	55.0 (54.0–56.0)	11.3 (10.5–12.0)
Noncitizen, Asian non-Hispanic	75.9 (74.7–77.1)	9.8 (8.9–10.7)
Noncitizen, other non-Hispanic race/ethnicity	57.3 (55.7–58.9)	12.1 (10.7–13.5)

a Primary eating and drinking consists of the total amount of time during the diary day that the respondent spent in eating and drinking as a primary activity. Secondary eating is the sum of all time during the diary day that the respondent spent eating during a primary activity. Multivariate linear regression models adjusted for age, sex, marital status, presence of children younger than 18 years in the household, education level, employment status, family income, metropolitan residence, region of country, and perceived health.

Although the magnitude varied, the pattern of results for the racial/ethnic identification of immigrants was consistent across immigrant status. Asian non-Hispanic immigrants spent the most time on primary eating and drinking, followed by immigrants who were White, non-Hispanic; Hispanic; other non-Hispanic race/ethnicity; and Black, non-Hispanic. For secondary eating time, noncitizens spent, on average, 11.1 minutes (95% CI, 10.4–11.8) per day compared with 12.2 minutes (95% CI, 11.6–12.8) for naturalized citizens and 12.9 minutes (95% CI, 12.7–13.0) for US-born respondents. We found a significant difference in secondary eating time between noncitizens and US-born respondents but found no significant difference between noncitizens and naturalized citizens or between US-born respondents and naturalized citizens. We found few significant differences by immigrant status and race/ethnicity for secondary eating time. Among US-born respondents and naturalized citizens, Asian non-Hispanic immigrants spent less secondary eating time per day than immigrants who reported other non-Hispanic race/ethnicity. Among noncitizens, we found no significant differences by race/ethnicity for secondary eating time.

Results from multivariate linear regression indicated that immigrants who had lived in the United States 5 years or less spent 69.7 (95% CI, 68.1–71.3) minutes per day in primary eating and drinking time, those who lived in the United States for 6 to 10 years spent 67.9 (95% CI, 66.4–69.3) minutes, those who lived in the United States for 11 to 15 years spent 63.6 (95% CI, 62.1–65.1) minutes, and those who lived in the United States for more than 15 years spent 63.6 (95% CI, 63.0–64.2) minutes (Table 3). Therefore, we found a difference of 6 minutes per day between immigrants who had lived in the United States 5 years or less and immigrants who had lived in the US for at least 15 years, which when assessed cumulatively results in an approximately 180-minute-per-month difference in primary eating time. For time per day spent in secondary eating, immigrants who lived in the United States for 5 years or less spent 5.5 (95% CI, 4.8–6.3) minutes, those who lived in the United States for 6 to 10 years spent 9.7 (95% CI, 8.7–10.7) minutes, those who lived in the United States for 11 to 15 years spent 8.4 (95% CI, 7.7–9.1) minutes, and those who lived in the United States more than 15 years spent 12.6 (95% CI, 12.2–13.1) minutes. (Full results from the regression analysis are available on request from the corresponding author.) 

**Table 3:** Adjusted Mean Number of Minutes Per Day Spent in Primary Eating and Drinking and Secondary Eating Activity Among Immigrants Aged 19 Years or Older, by Length of Residence in the United States, American Time Use Survey, 2016^a^

Years Lived in the United States	Primary Eating and Drinking	Secondary Eating
	Mean (95% CI)	Mean (95% CI)
≤5	69.7 (68.1–71.3)	5.5 (4.8–6.3)
6–10	67.9 (66.4–69.3)	9.7 (8.7–10.7)
11–15	63.6 (62.1–65.1)	8.4 (7.7–9.1)
>15	63.6 (63.0–64.2)	12.6 (12.2–13.1)

a Primary eating and drinking consists of the total amount of time during the diary day that the respondent spent in eating and drinking as a primary activity. Secondary eating is the sum of all time during the diary day that the respondent spent eating during a primary activity. Multivariate linear regression models adjusted for age, sex, marital status, presence of children younger than 18 years in the household, education level, employment status, family income, metropolitan residence, region of country, and perceived health.

## Discussion

We examined the association between time spent in primary and secondary eating and immigrant status, race/ethnicity, and years lived in the United States and found that noncitizens spent more time per day in primary eating and less time per day in secondary eating than US-born individuals. We also found that years lived in the United States was negatively associated with time spent eating and drinking. The pattern of results for race/ethnicity and primary eating time was consistent across immigrant status. Asian non-Hispanic immigrants spent the most time on primary eating and drinking, followed by White non-Hispanic, Hispanic, other race/ethnicity, and Black non-Hispanic respondents. We found limited significant differences in time spent eating by immigrant status and race/ethnicity. Naturalized citizens and US-born respondents who reported their race/ethnicity as Asian, non-Hispanic spent less time in secondary eating compared with other racial/ethnic immigrant groups. However, we found no significant differences to report by race/ethnicity for secondary eating time among noncitizen immigrants.

Acculturation has been measured by citizenship, language, and years spent in the United States, all of which are associated with health behaviors (22–25,29). Moreover, more time spent in the United States is associated with better access to health care and lower health care use, which may further exacerbate health disparities related to acculturation for recently arrived immigrants (30). Immigrants may adopt mainstream American health behaviors over time, gradually emulating behaviors of US-born individuals (16,27,29). For example, a higher number of years living in the United States among immigrants has been associated with a decline in consumption of healthy foods such as fruits and vegetables (23,24,27,29). This decline in consumption has also been linked to language use in the home, with ethnic groups that speak mostly English at home consuming more energy-dense, non-homemade meals compared with groups that speak the language from their country of origin at home (26). The mounting evidence suggests that US culture may influence immigrants to adopt unhealthy behaviors and, by extension, be at greater risk of chronic disease (16,22,29).

Our study has limitations. Although time diaries have been validated as a measure for time use and the American Time Use Survey is widely used as a source of information on how people spend their time, this measure is limited to a given 24-hour period; if the time diary was collected at a different point, the activities of the person surveyed may differ (28). Moreover, time diaries are cross-sectional, self-reported data rather than directly measured data of a prospective cohort. ATUS data did not include geographic identifiers to link them to states and counties, which would have enabled us to control for state or county characteristics, an important contextual variable to consider when measuring health effects. Another limitation is that the data did not include a measure on language, which is a well-established measure of acculturation. Finally, the data are a diary of time use rather than a detailed food diary, which may have been helpful for interpreting the time spent in primary and secondary eating time.

In conclusion, we found that time spent eating varied by immigrant status and length of residence in the United States. We found limited support for the hypothesis that differences in eating time by immigrant status and race/ethnicity would exist. Immigrants may be shifting toward eating behavior that is characterized by less time spent food shopping, preparing meals, and food consumption as a primary activity and more time spent eating while engaged in other activities such as watching television. Future research should assess randomized controlled interventions that use culturally tailored approaches for immigrants to increase time spent in meal preparation and consumption of freshly prepared foods and reduce time spent in secondary eating of pre-prepared or processed foods. Nutrition and diet-related interventions should tailor interventions by years lived in the United States and the race/ethnicity of immigrant groups. Public health policy and programs should address the negative impact that acculturation has on eating behaviors and the heightened risk for obesity and other diet-related diseases in the United States.

## References

[1] Zick CD , Stevens RB , Bryant WK . Time use choices and healthy body weight: a multivariate analysis of data from the American Time Use Survey. Int J Behav Nutr Phys Act 2011;8(1):84. 10.1186/1479-5868-8-84 21810246 PMC3199736

[2] Zeballos E , Restrepo B . Adult eating and health patterns: evidence from the 2014–2016 Eating and Health module of the American Time Use Survey. Washington (DC): United States Department of Agriculture, Economic Research Service; 2018.

[3] Zick CD , Stevens RB . Trends in Americans’ food-related time use: 1975–2006. Public Health Nutr 2010;13(7):1064–72. 10.1017/S1368980009992138 19943999

[4] Monsivais P , Aggarwal A , Drewnowski A . Time spent on home food preparation and indicators of healthy eating. Am J Prev Med 2014;47(6):796–802. 10.1016/j.amepre.2014.07.033 25245799 PMC4254327

[5] Fiese BH . Time allocation and dietary habits in the United States: Time for re-evaluation? Physiol Behav 2018;193(Pt B):205–8. 10.1016/j.physbeh.2018.02.040 29474838

[6] Nguyen BT , Powell LM . The impact of restaurant consumption among US adults: effects on energy and nutrient intakes. Public Health Nutr 2014;17(11):2445–52. 10.1017/S1368980014001153 25076113 PMC10282383

[7] Guthrie JF , Lin BH , Frazao E . Role of food prepared away from home in the American diet, 1977-78 versus 1994-96: changes and consequences. J Nutr Educ Behav 2002;34(3):140–50. 10.1016/S1499-4046(06)60083-3 12047838

[8] Poti JM , Mendez MA , Ng SW , Popkin BM . Is the degree of food processing and convenience linked with the nutritional quality of foods purchased by US households? Am J Clin Nutr 2015;101(6):1251–62. 10.3945/ajcn.114.100925 25948666 PMC4441809

[9] Smith LP , Ng SW , Popkin BM . Trends in US home food preparation and consumption: analysis of national nutrition surveys and time use studies from 1965-1966 to 2007-2008. Nutr J 2013;12(45):45. 10.1186/1475-2891-12-45 23577692 PMC3639863

[10] Bowman SA . Television-viewing characteristics of adults: correlations to eating practices and overweight and health status. Prev Chronic Dis 2006;3(2):A38. 16539779 PMC1563980

[11] Pearson N , Biddle SJ . Sedentary behavior and dietary intake in children, adolescents, and adults. A systematic review. Am J Prev Med 2011;41(2):178–88. 10.1016/j.amepre.2011.05.002 21767726

[12] Briefel RR , Johnson CL . Secular trends in dietary intake in the United States. Annu Rev Nutr 2004;24(1):401–31. 10.1146/annurev.nutr.23.011702.073349 15189126

[13] Zamora-Kapoor A , Sinclair K , Nelson L , Lee H , Buchwald D . Obesity risk factors in American Indians and Alaska Natives: a systematic review. Public Health 2019;174:85–96. 10.1016/j.puhe.2019.05.021 31326761

[14] Osei-Assibey G , Kyrou I , Adi Y , Kumar S , Matyka K . Dietary and lifestyle interventions for weight management in adults from minority ethnic/non-White groups: a systematic review. Obes Rev 2010;11(11):769–76. 10.1111/j.1467-789X.2009.00695.x 20059708

[15] Fitzgibbon ML , Tussing-Humphreys LM , Porter JS , Martin IK , Odoms-Young A , Sharp LK . Weight loss and African-American women: a systematic review of the behavioural weight loss intervention literature. Obes Rev 2012;13(3):193–213. 10.1111/j.1467-789X.2011.00945.x 22074195 PMC3288708

[16] Popkin BM . The nutrition transition and obesity in the developing world. J Nutr 2001;131(3 Suppl 3):871S–3S. 10.1093/jn/131.3.871S 11238777

[17] Ngongalah L , Rankin J , Rapley T , Odeniyi A , Akhter Z , Heslehurst N . Dietary and physical activity behaviours in African migrant women living in high income countries: a systematic review and framework synthesis. Nutrients 2018;10(8):1017. 10.3390/nu10081017 30081522 PMC6115772

[18] Ayala GX , Baquero B , Klinger S . A systematic review of the relationship between acculturation and diet among Latinos in the United States: implications for future research. J Am Diet Assoc 2008;108(8):1330–44. 10.1016/j.jada.2008.05.009 18656573 PMC3727241

[19] Delavari M , Sønderlund AL , Swinburn B , Mellor D , Renzaho A . Acculturation and obesity among migrant populations in high income countries—a systematic review. BMC Public Health 2013;13:458. 10.1186/1471-2458-13-458 23663279 PMC3654930

[20] Rosenmöller DL , Gasevic D , Seidell J , Lear SA . Determinants of changes in dietary patterns among Chinese immigrants: a cross-sectional analysis. Int J Behav Nutr Phys Act 2011;8(1):42. 10.1186/1479-5868-8-42 21592378 PMC3118129

[21] Lesser IA , Gasevic D , Lear SA . The association between acculturation and dietary patterns of South Asian immigrants. PLoS One 2014;9(2):e88495. 10.1371/journal.pone.0088495 24558396 PMC3928252

[22] Pérez-Escamilla R . Acculturation, nutrition, and health disparities in Latinos. Am J Clin Nutr 2011;93(5):1163S–7S. 10.3945/ajcn.110.003467 21367946 PMC3076661

[23] Stimpson JP , Urrutia-Rojas X . Acculturation in the United States is associated with lower serum carotenoid levels: Third National Health and Nutrition Examination Survey. J Am Diet Assoc 2007;107(7):1218–23. 10.1016/j.jada.2007.04.008 17604755

[24] Marín-Guerrero AC , Rodríguez-Artalejo F , Guallar-Castillón P , López-García E , Gutiérrez-Fisac JL . Association of the duration of residence with obesity-related eating habits and dietary patterns among Latin-American immigrants in Spain. Br J Nutr 2015;113(2):343–9. 10.1017/S0007114514003614 25418887

[25] Hamermesh DS , Trejo SJ . How do immigrants spend their time? The process of assimilation. J Popul Econ 2013;26(2):507–30. 10.1007/s00148-012-0440-x 24443631 PMC3891674

[26] Langellier BA , Brookmeyer R , Wang MC , Glik D . Language use affects food behaviours and food values among Mexican-origin adults in the USA. Public Health Nutr 2015;18(2):264–74. 10.1017/S1368980014000287 24698136 PMC4469614

[27] Sproesser G , Ruby MB , Arbit N , Akotia CS , Alvarenga MDS , Bhangaokar R , Understanding traditional and modern eating: the TEP10 framework. BMC Public Health 2019;19(1):1606. 10.1186/s12889-019-7844-4 31791293 PMC6889524

[28] American Time Use Survey. https://www.bls.gov/tus/home.htm. Accessed October 7, 2020.

[29] Satia-Abouta J , Patterson RE , Neuhouser ML , Elder J . Dietary acculturation: applications to nutrition research and dietetics. J Am Diet Assoc 2002;102(8):1105–18. 10.1016/S0002-8223(02)90247-6 12171455

[30] Derose KP , Bahney BW , Lurie N , Escarce JJ . Review: immigrants and health care access, quality, and cost. Med Care Res Rev 2009;66(4):355–408. 10.1177/1077558708330425 19179539

